# Postural Variabilities Associated with the Most Comfortable Sitting Postures: A Preliminary Study

**DOI:** 10.3390/healthcare9121685

**Published:** 2021-12-06

**Authors:** Yi-Lang Chen, You-Chun Chan, Li-Peng Zhang

**Affiliations:** Department of Industrial Engineering and Management, Ming Chi University of Technology, New Taipei 24301, Taiwan; m09218011@mail2.mcut.edu.tw (Y.-C.C.); m09218004@mail2.mcut.edu.tw (L.-P.Z.)

**Keywords:** sitting comfort, perception, chair type, postural variability, global joint angle

## Abstract

This study examined postural variabilities based on the self-perceived most comfortable postures of 12 participants (six men and six women) when sitting on three commonly used types of chairs (a stool, computer chair, and gaming chair). Participants’ global joint angles were recorded and analyzed. Of the chairs studied, the stool was not adjustable, but the computer and gaming chairs were moderately and highly adjustable, respectively. During the test, participants were encouraged to adjust the chairs until they perceived that the most comfortable posture had been reached. The results demonstrated that in a sitting position perceived to be comfortable, the participants’ postural variabilities with respect to global joint angle, calculated from five repetitions, were unexpectedly high for all three chair types, at approximately 9.4, 10.2, and 11.1° for head inclination, trunk angle, and knee angle, respectively. The average differences in range for each joint angle among the three chair types were relatively low, with all values within 3°. The result also showed that gender (*p* < 0.01) and chair type (*p* < 0.001) significantly affected trunk angle, whereas these variables did not affect head inclination or knee angle (*p* > 0.05). The preliminary results observed unexpectedly high variabilities in sitting posture when the participants sat at a posture that they perceived to be the most comfortable. The findings also indicated an inherent difference in comfortable sitting posture between genders; women tend to extend their trunk backward more than men. For permanent use with only an initial adjustment and memory-aided seat design, designers should minimize the loads that are borne by body parts over a prolonged period due to an unchanging sitting posture.

## 1. Introduction

In response to changes in work and lifestyle, sitting posture has become increasingly important for people, including for office work, computer gaming, and driving. People spend most of their time sitting, whether at work or at play [1], bringing with it body discomfort, pain, and even injury, particularly to the neck and shoulders and lower back [2,3].

The correct, or optimal sitting posture remains widely debated. An erect sitting posture is generally adopted in daily life and is attached to the socially constructed notion of an optimal posture [4]. The optimal posture is rarely determined by body strain and comfort, and many researchers have assessed sitting posture from an ergonomic perspective. For example, a good sitting posture should have a lordotic lumbar spinal curve similar to standing [5,6], and spinal pain can be caused by a flexed sitting posture [7]. However, a flexed posture is commonly adopted in daily sitting [8,9], and this habitual sitting posture has been considered as a mid-range position involving more flexion than other sitting postures [10,11]. Experts in ergonomics who favor an erect sitting posture [12,13] note that it may lead to increased levels of fatigue resulting from increased muscle activation compared with the habitual sitting posture; but scientific evidence that any specific posture causes spinal pain is lacking [14].

In the literature, what constitutes an optimal sitting posture remains questionable, with no consensus on a single correct posture [15]. Any posture, (e.g., erect or slumped, lordotic or kyphotic) if maintained for a prolonged period, could lead to discomfort and even injury [16]. Furthermore, individuals may respond differently to various chair types, and the factors that influence sitting posture are still unknown [17]. Clinically, the corrective postural interventions and support commonly used to manage spinal pain are based on an assumption that postural variations from the optimal position may cause lower back pain (LBP) [18]. A popular theory is that good sitting habits involve frequent postural adjustments [6]. This implies that when people are sitting in a position that they perceive to be comfortable, the posture is frequently changed. Studies have also indicated that low-force activities, such as sitting, reduce trunk motor variability, which is associated with increased muscle fatigue and decreased endurance and may thus cause LBP [19,20].

Although methods for changing sitting posture include adjustable seats and active postural changes by sitters, not all chairs are equipped with adjustment devices. Studies have also demonstrated that even if an adjustment mechanism is present, it is rarely used, and users tend to adjust the seat the first time they use it [21]. This may be because sitters feel less need to readjust the seat once they perceive that it is comfortable. In this regard, the consistency of the seat user’s subjectively perceived most comfortable posture may become crucial to examining if a comfortable sitting posture exists.

Whereas seat users agree on the definition of discomfort and the pain involved in sitting in a bad chair, the comfort or the positive aspects have been difficult to define. Helander [22], thus, offered an operational definition of sitting comfort including a sense of relaxation and relief. This may imply that comfort makes the sitters perceive the least stress on the body when sitting. In the evaluation of seats, different methods have been used to measure sitting comfort, such as anthropometry, subjective assessment, and objective measurements. In general, questionnaires are often developed to assess the comfort/discomfort levels for comparison purposes [23,24,25], whereas the anthropometric and biomechanical evaluations are used for chair designs [26,27,28] and load estimated on body regions [29,30], respectively. Body joint angle recoding is one of the methods for connecting the sitting posture and the comfort. Kee and Karwowski [24] proposed an index of the joint angles of isocomfort in sitting and standing based on perceived comfort ratings for static joint postures and indicated that postures maintained for 60 s cause greater discomfort for the hip joint than for the other joints studied, and less discomfort for the elbow than for the other joints. Korakakis et al. [12] discovered that a relatively large range of movement occurred in head-tilt angles from one day to the next when a neutral sitting posture was adopted, with a difference of 6.0°. The difference was averaged across 26 participants, with larger differences in postures also being observed. However, subjective and objective parameters are usually not independent, thus sitting postures or other physiological indicators that cause the sitters more comfortable could be determined by exploring their relationship [23,29].

Studies have examined what parameters of chair design affect (and how they affect) the sitters’ discomfort levels, and researchers have thus attempted to design a chair that is more comfortable for sitters [2,3,27,30]. In this regard, the optimal, habitual, and correct sitting postures have been investigated. However, no research has focused on the variability in comfortable sitting postures as determined by sitters. Currently, some seats, such as those in cars, are equipped with personal memory-aid adjustment devices. The premise of these devices is that individuals tend to perceive comfort in an identical posture, but this remains undetermined. This work, therefore, evaluated the postural variabilities with respect to joint angles between three commonly used chairs and between genders based on the self-perceived most comfortable postures.

## 2. Materials and Methods

### 2.1. Participants

The experiment recruited 12 healthy young participants (six men and six women) for the tests, the relatively small sample size was because of its preliminary nature. Individuals with medical histories of musculoskeletal disorders were excluded. Each participant was informed of details of the study in general and the experimental procedures in particular. Participants’ age, height, and body mass were collected prior to the experiment. The experiment was performed in accordance with the 2013 World Medical Association Declaration of Helsinki. The Ethics Committee of National Taiwan University approved the experimental procedures. All participants provided written consent prior to the experiment and were remunerated for their time. The participants were representative of the general Taiwanese population with respect to their anthropometric characteristics [31].

### 2.2. Chair Types

**Figure 1:**
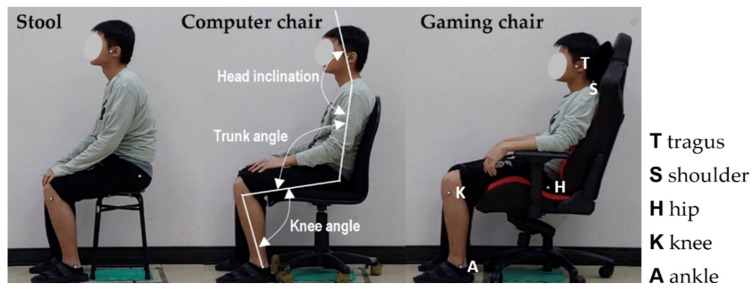
Three types of chairs and global joint angles examined in the test.

The three chair types examined in the test included a stool (X + Y Fashion Boutique Furniture, Tainan, Taiwan), a computer chair (SA03G, COSMOS, Taipei, Taiwan), and a gaming chair (ISKUR, RZ38-02770100-R3U1, Razer, Singapore), as shown in Figure 1. Although the stool could not be adjusted, the seat height of the computer and gaming chairs was adjustable, and these chairs were also equipped with a resilient (computer chair) and adjustable back support (gaming chair, range: 90°–140°). In addition, the gaming chair’s armrests were adjustable in terms of height and horizontal rotation. The chairs adopted in the test are commonly used by Taiwanese people in the workplace or in everyday life.

### 2.3. Global Joint Angle Measurements

Prior to the test, the experimenters attached five reflective markers with a 2-cm diameter to the participants’ left tragus, shoulder (the acromial shelf), hip (the greater trochanter), knee (the lateral epicondyle), and ankle (the lateral malleolus) joints. Once the participants had adjusted the chair to support a posture that they were most comfortable with, the global joint angles (head inclination, HI; trunk angle, TA; knee angle, KA) in the sagittal plane were recorded (Figure 1). A MacReflex motion analysis system (Qualisys, Göteborg, Sweden) was positioned approximately 5 m to the left-lateral side of the participant and perpendicular to the participant’s sagittal plane to record the 2D marker positions. The motion analysis system was also used to determine the sagittal joint angles for each recording by researchers YCC and LPZ.

### 2.4. Experimental Design and Procedure

In the test, participants were asked to wear light clothing to clearly identify each joint position. All the experiments, including the three chair types and the five repeated trials for each chair, were completed within 3 h randomly for each participant. Consequently, a total of 180 test combinations (12 participants × 3 chair types × 5 repetitions) were implemented for the HI, TA, and KA measurements. During the test, participants were encouraged to adjust the chair using the attached knobs and levers until it supported a posture that they perceived to be the most comfortable. All participants were given instruction as follows:

*In this experiment we would like to collect the most comfortable sitting posture. Please adjust your sitting posture as much as possible. We encourage you to use the seat adjustment devices and move your body joint angles until the most comfortable posture you perceive. Be sure that this posture for the seat makes you feel the most relaxed and the stresses on the body parts are minimum. If the most comfortable posture is achieved, please say YES.*

Once the posture was determined by the participants, it was recorded through the motion analysis system for further analysis (researchers YLC and YCC). Before each trial was performed, the seat parameters were adjusted randomly by an experimenter except for the stool. The resting time was set at a minimum of 10 min between trials. The design was considered to minimize any interference caused by extreme daily activities (e.g., exercise or prolonged sitting) if the tests were performed on alternate days. When resting, participants were allowed to undertake light activities, such as standing, walking, or sitting (the chair differed from the three chair types examined in the test).

### 2.5. Statistical Analysis

The data collected in the experiment were analyzed using SPSS 23.0 statistical software (IBM Corp., Armonk, NY, USA), at a significance level of α = 0.05 for all tests. The descriptive statistics of the mean, range (i.e., differences between maximum and minimum values), and standard deviation of each joint angle, calculated from the five repeated trials for each chair type, were calculated. The effects of independent variables (gender and chair type) were examined using a two-way repeated-measures analysis of variance (ANOVA), and the Duncan multiple range test (MRT) was used for multiple comparisons. Moreover, a one-way ANOVA and the Duncan MRT were conducted on mean and ranged joint angles for each gender. A power value was used to examine if the effect size of any significant independent variable was satisfactory (i.e., power ≥ 0.8) as suggested by Cohen [32]. Beforehand, the Shapiro-Wilk test was used to verify the compliance of numerical variables with the normal distribution, while the Levene’s test was used to verify the homogeneity of variances.

## 3. Results

The mean (standard deviation) age, height, and body mass for the male participants were 23.1 (2.4) years, 171.8 (3.5) cm, and 67.6 (6.8) kg, respectively, and those for the female participants were 22.7 (2.1) years, 160.4 (3.8) cm, and 53.1 (6.0) kg, respectively. Through Shapiro–Wilk test, the joint angle data collected in the study were normally distributed (all *p* > 0.05, Range: 0.092–0.863) meanwhile Levene’s test showed the data were homogenous (all *p* > 0.05, Range: 0.105–0.798). The results for the two-way ANOVA of means and ranges calculated from the five test repetitions for the three joint angles are shown in Table 1 and Table 2, respectively. Gender (*p* < 0.01, power = 0.851) and chair type (*p* < 0.001, power = 0.954) had significant effects on mean TA, whereas these two variables did not affect the range of any joint angle. Table 3 presents the one-way ANOVA results of chair type on joint angle for each gender. As shown in the table, the mean TA was influenced by both males (*p* < 0.05, power = 0.741) and females (*p* < 0.01, power = 0.820), but the mean HI was only influenced by females (*p* < 0.05, power = 0.738).

**Figure 2:**
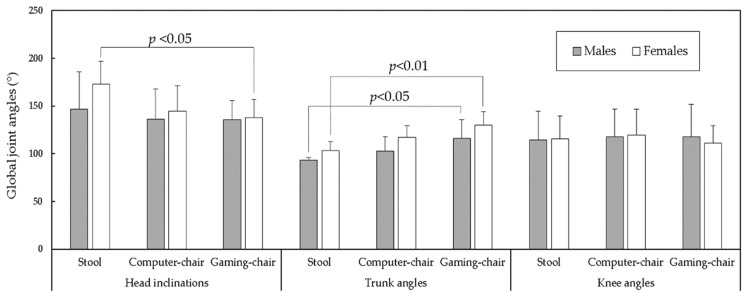
Comparisons of mean joint angles for various test combinations and the Duncan MRT test results.

**Figure 3:**
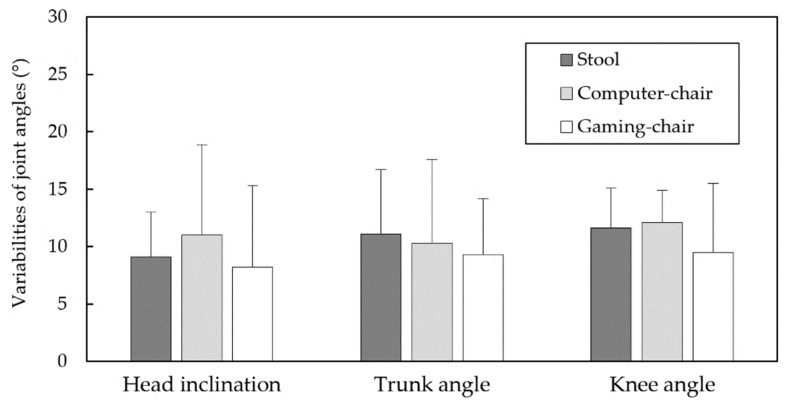
Comparisons of variabilities of joint angles for various test combinations.

Figure 2 presents the joint angles for each gender when sitting on the three types of chairs. The Duncan MRT results demonstrated that, regardless of gender, TA was higher when sitting in a gaming chair than when sitting on a stool. By contrast, when the female participants sat in a gaming chair, their HI was lower than when on a stool. Figure 3 shows the variabilities of the joint angles in all test combinations. Although no difference was found for any comparison pair, the variabilities of each joint angle for the three chair types were all greater than 8°, at approximately 9.4, 10.2, and 11.1° for HI, TA, and KA, respectively. However, the average difference in range of each joint angle between the three chairs was relatively low, at less than 3°.

## 4. Discussion

Although previous studies have explored the optimal, correct, and habitual sitting postures, they have not examined the postural variabilities associated with the posture that an individual perceives to be the most comfortable. This preliminary study found that variabilities in the global joint angle for the most comfortable sitting posture were unexpectedly high. More caution should thus be exercised in the seats with only an initial adjustment and memory-aided seat design aimed at achieving a comfortable sitting posture for permanent use. However, why the variabilities of joint angles were unexpectedly high observed in the result remains unknown and needs clarification in the future.

**Figure 4:**
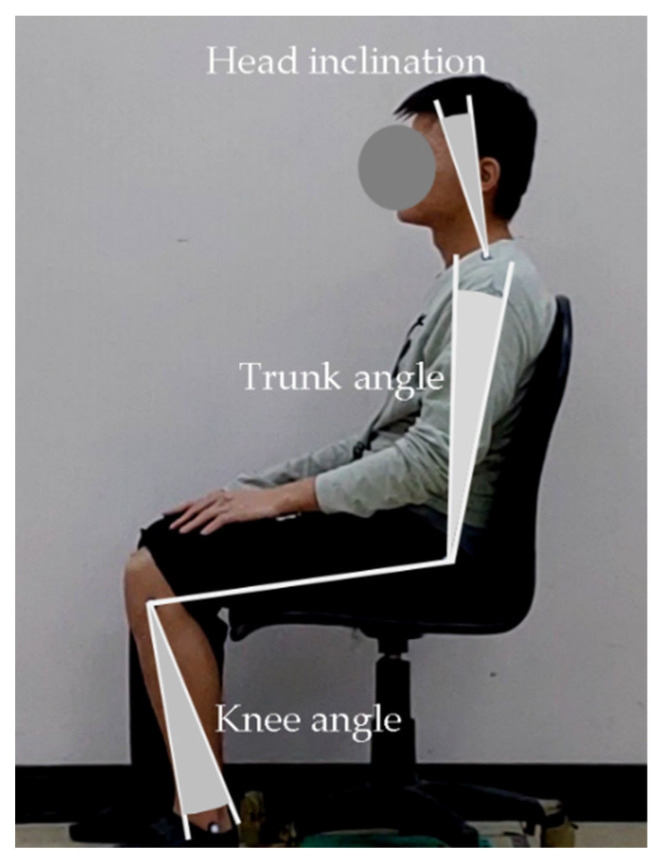
Ranges of the angle variations for each joint based on the five repeated trials.

Studies have suggested that muscle tone and the corresponding body posture are controlled by the multiplicity of nervous system pathways [33], which cause postural variance through both increased and decreased muscle tone. In the results of this investigation, the variabilities in global joint angles, calculated from the five repeated trials on HI, TA, and KA using three chair types and based on participants’ perception of comfort, exceeded 8° for all test combinations, with averages of 9.4, 10.2, and 11.1°, respectively (Figure 4). Korakakis et al. [12] discovered that a relatively large range of movement occurred at head-tilt angles from one day to the next when a neutral sitting posture was adopted, with a difference of 6.0°. The results were more varied than those of Korakakis et al., suggesting that the consistency of people’s perceptions of their most comfortable sitting posture may be unreliable and may be affected posture-by-posture. In addition, no significant difference in variations between sitting postures was present between gender, chair type, or joint (Table 2), and the average differences in the range of each joint angle between the three chair types were relatively low, with all values within 3°. Claeys et al. [34] observed large variability in sagittal spinal posture between individuals, without the existence of any optimal sagittal posture; the same might be also true for the most comfortable sitting posture determined by an individual. This study, therefore, suggests that for permanent seat use after only an initial adjustment and memory-aided seat design, cumulative body part loads caused by unchanged posture may be inappropriate, particularly in the case of prolonged sitting. However, whether the finding of this work could be completely generalized to the memory-aided car seats requires further examination.

Researchers have speculated that people only occasionally consider their own posture [7]. Scholarly evidence suggests that habitual sitting posture usually favors a more flexed posture than other upright postures [10,35]. Studies have also indicated that the self-perceived optimal posture is more upright than one’s habitual posture or that during spontaneous sitting tasks [10,35]. In summary, the habitual sitting posture usually features greater flex than upright and optimal postures. By contrast, Korakakis et al. [9] recently found that self-perceived optimal posture was significantly more extended than habitual sitting posture in the majority of spinal regions. However, differences exist between studies. Sitting postures that match the natural shape of the spine, which are similar to stereotypes of what optimal posture is and appear to be comfortable or relaxed without excessive muscle tone, have often been deemed by scholars to be advantageous [12,13], suggesting that the most comfortable sitting posture is similar to a habitual sitting posture, with the trunk being relatively flexed forward. However, the results of the present study are inconsistent with those of previous publications, with every participant determining their own sitting posture to assume the most comfortable posture. A possible explanation is that different seat characteristics (e.g., having a backrest) affected the habitual posture that was adopted. According to the results, the higher the degree of adjustment or seat comfort, the more the trunk extended backward. Although many studies believe that sitting posture affects the posture of the spine, Korakakis et al. [9] found that the lack of significant differences in the different spinal regions between postures may be attributed to the sitting posture being primarily driven by the position of the pelvis and not the lumbar spine [36,37]. In the present study, the trunk angle was based on the position of the shoulders, greater trochanter, and knees (Figure 1), and the spinal movement during sitting was thus not discussed.

The results indicate that TA did not only significantly differ between gender (Table 1), men and women also significantly differed individually in TA between chair types (Table 3). Dunk and Callaghan [17] reported that regardless of the chair used or the task performed, TAs exhibited significantly greater flex in men than in women; by contrast, other researchers have observed women tend to adopt a more upright habitual sitting posture than men [34,38]. In this test, the TA for a comfortable sitting posture on a stool was the smallest (men: 93.1°, women: 103.2°), followed by the computer chair (men: 102.8°, women: 117.0°) and the gaming chair (men: 115.9°, women: 129.9°); however, the larger TA also resulted in a smaller HI for women (stool: 172.8°, gaming chair: 138.0°; *p* < 0.05), as indicated in Figure 2. The larger extended trunk positions for the computer and gaming chairs may be because these chairs had a backrest. The factors affecting the gender-specific postural differences may result from gender differences in pelvic geometry [39], motor activation patterns [40], different spinal loadings [17], and psychosocial status [41]. Compared with previous studies focusing on habitual or optimal sitting postures, relatively little information is available regarding the most comfortable sitting posture. The preliminary results obtained in this test should be verified in further studies.

This study has several limitations. First, it was limited by the sample size of the participants. The primary weakness of this preliminary study was that a relatively small sample (six male and six female university students) was recruited in the test. In the results, some effect sizes (power values) for the analyses were more than the criteria (i.e., 0.8), however, some were close to but less than 0.8. This small sample definitely alters the robustness of the results and may limit the generalization of the findings. More samples and other populations should be included for future investigation. Second, only global joint angles of the participants were recorded in the test and the spinal movement during sitting was not examined. Understanding the variation of the spinal posture when sitting (e.g., lordosis or kyphosis) may have also an impact especially on the comfort of the lower back region. In addition, sitting comfort was determined only by participants’ subjective perception in the experiment, assessments using other quantitative protocols may provide more valuable information for this topic.

## 5. Conclusions

This study was first to preliminarily examine the variability in comfortable sitting postures as determined by sitters, and the findings can serve as a reference for the design of adjustable seats. In the analyses, unexpectedly high variabilities in sitting posture were observed when the participants sat at a posture that they perceived to be the most comfortable, regardless of gender, chair type, and joint. The seat designs based on the concept of unchanging sitting posture should be more cautious.

## Figures and Tables

**Table 1:** Two-way ANOVA results for mean joint angles calculated from 5 repeated trials.

	Head Inclination		Trunk Angle		Knee Angle	
Variables	F	*p*	F	*p*	F	*p*
Gender	1.70	0.202	8.05	<0.01	0.02	0.892
Chair type	2.32	0.116	10.10	<0.001	0.08	0.925
Gender × chair type	0.58	0.566	0.09	0.914	0.10	0.906

**Table 2:** Two-way ANOVA results for the ranges of joint angles calculated from 5 repeated trials.

	Head Inclination		Trunk Angle		Knee Angle	
Variables	F	*p*	F	*p*	F	*p*
Gender	0.67	0.418	1.95	0.172	0.04	0.838
Chair type	1.29	0.290	0.23	0.795	0.60	0.553
Gender × chair type	1.52	0.235	0.65	0.528	0.557	0.579

**Table 3:** One-way ANOVA results for chair type in relation to joint angle for each gender.

	Head Inclination		Trunk Angle		Knee Angle	
**Mean Joint Angle**	**F**	***p***	**F**	***p***	**F**	***p***
Males	0.24	0.793	3.62	<0.05	0.02	0.979
Females	3.61	<0.05	7.31	<0.01	0.63	0.405
**Joint Angle Range**	**F**	***p***	**F**	***p***	**F**	***p***
Males	1.50	0.254	0.52	0.605	0.61	0.557
Feamles	1.12	0.350	0.25	0.785	0.57	0.578
